# Patient-derived organoids support radiotherapy response and recurrence risk prediction in esophageal squamous cancer

**DOI:** 10.1016/j.isci.2026.117100

**Published:** 2026-08-10

**Authors:** Eunji Jeong, Jong Yun Baek, Sang-Yun Lee, Yongmun Choi, Bosung Ku, Dan Bi Park, Man Ki Chung, Dong Woo Lee, Dongryul Oh

**Affiliations:** 1 Central R&D Center, Medical & Bio Decision (MBD) Co., Ltd, Suwon, Republic of Korea; 2 Department of Radiation Oncology, Samsung Medical Center, Sungkyunkwan University School of Medicine, Seoul, Republic of Korea; 3 Graduate School of New Drug Discovery and Development, Chungnam National University, Daejeon, Republic of Korea; 4 Department of Biomedical Engineering, Gachon University, Seongnam, Republic of Korea

**Keywords:** esophageal cancer, cancer organoids, precision medicine, prediction model of radiotherapy

## Abstract

Predicting individual responses to radiotherapy remains a major challenge in esophageal cancer. This study evaluated an cancer organoid-based diagnosis and reactivity prediction (CODRP) approach to estimate pathological response and recurrence risk after neoadjuvant chemoradiotherapy (NCRT) in esophageal squamous cell carcinoma. Organoids were established from endoscopic biopsy specimens and irradiated with doses of 2, 4, or 8 Gy. Growth inhibition-based area under the curve (GI-AUC) and cell growth rates were quantified and integrated with the clinical cancer stage to generate the CODRP index. Compared with single-parameter models, the CODRP showed improved apparent predictive performance for pathological response prediction in this feasibility cohort (sensitivity, 87.5%; specificity, 100%). Recurrence-free survival was significantly higher in the CODRP-classified radiation-sensitive group (71.43%) than in the radiation-resistant group (10%) (*p* = 0.0103). These findings support the feasibility of applying CODRP to radiotherapy response and recurrence risk prediction in esophageal squamous cell carcinoma.

## Highlights

• Diagnostic biopsy PDOs enabled two-week CODRP RT response and recurrence prediction
• CODRP outperformed single metrics with 87.5% sensitivity and 100% specificity
• CODRP radiosensitive patients had higher 2-year RFS 71.43% vs 10%, p = 0.0103
• PDO-based multiparametric platform may guide pretreatment therapy in esophageal SCC

## Graphical abstract

**Figure:**
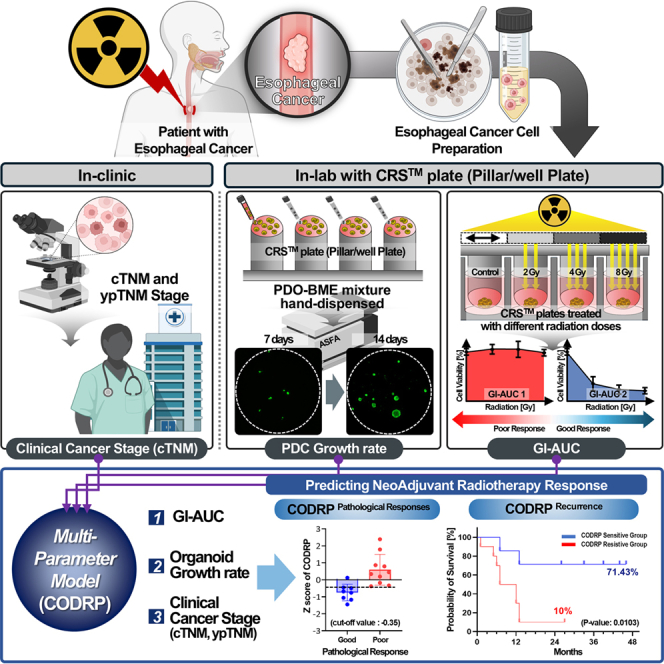

## Teaser

Biological sciences; Biocomputational method; Cancer

## Introduction

Esophageal carcinoma represents a significant global health burden, as the seventh most common malignancy and the sixth leading cause of cancer-related death worldwide.^1^^,^^2^ Two principal histological subtypes-squamous cell carcinoma (SCC) and adenocarcinoma (ADC)-differ in biology and geography, with SCC predominating in Eastern Asia.^3^^,^^4^ For locally advanced esophageal SCC, neoadjuvant chemoradiotherapy (NCRT) followed by surgery remains the standard of care, improving disease control and survival outcomes.^5^^,^^6^ However, only around 30 to 50 percent of patients experience pathological complete response (pCR), while others exhibit little improvement.^7^ This emphasizes the necessity for precise prognostic methods for personalized treatment strategies. Furthermore, pCR reflects high tumor sensitivity to NCRT and can inform subsequent treatment decisions; Millen et al. recently demonstrated that organoids derived from patients with head and neck cancer (HNC) could predict individual responses to radiotherapy.^8^^,^^9^ These findings suggest the potential for developing personalized treatment strategies in oncology. Notably, the predictive power of the organoid model was statistically significant only when the growth rate-based half-maximal inhibitory concentration (GR50) was used to classify the organoids into sensitive and resistant groups.

Previously, we established an organoid-based high-throughput screening platform to evaluate organoid viability across a range of radiation doses.^10^ The area under the radiation dose-response (AUC) was used as a predictive metric for radiotherapy outcomes in patients with cancer and basic research.^11^ In the present study, we observed that organoid growth varied significantly among patients, which affected the accuracy of the radiotherapy response predictions. To improve predictive performance, we incorporated multiple parameters, including growth inhibition-based AUC (GI-AUC), organoid growth rate, and clinical staging, into our model to predict the pathological response to NCRT and assess the risk of recurrence in esophageal cancer. Previous studies have introduced the cancer organoid-based diagnosis and reactivity prediction (CODRP) model as a multiparametric framework for predicting anticancer drug sensitivity using patient-derived organoids (PDOs).^12^ These approaches have demonstrated improved predictive performance compared to single-parameter metrics. However, the application of such multiparametric organoid-based models to radiotherapy response prediction remains largely unexplored. In this study, we evaluate the potential of the CODRP framework to predict radiation sensitivity, NCRT response, and recurrence risk in esophageal SCC using PDOs established from pretreatment biopsy samples.

## Results

### 3D-cultured cell lines on the cell radiation sensitivity (CRS™) plate

**Figure 1:**
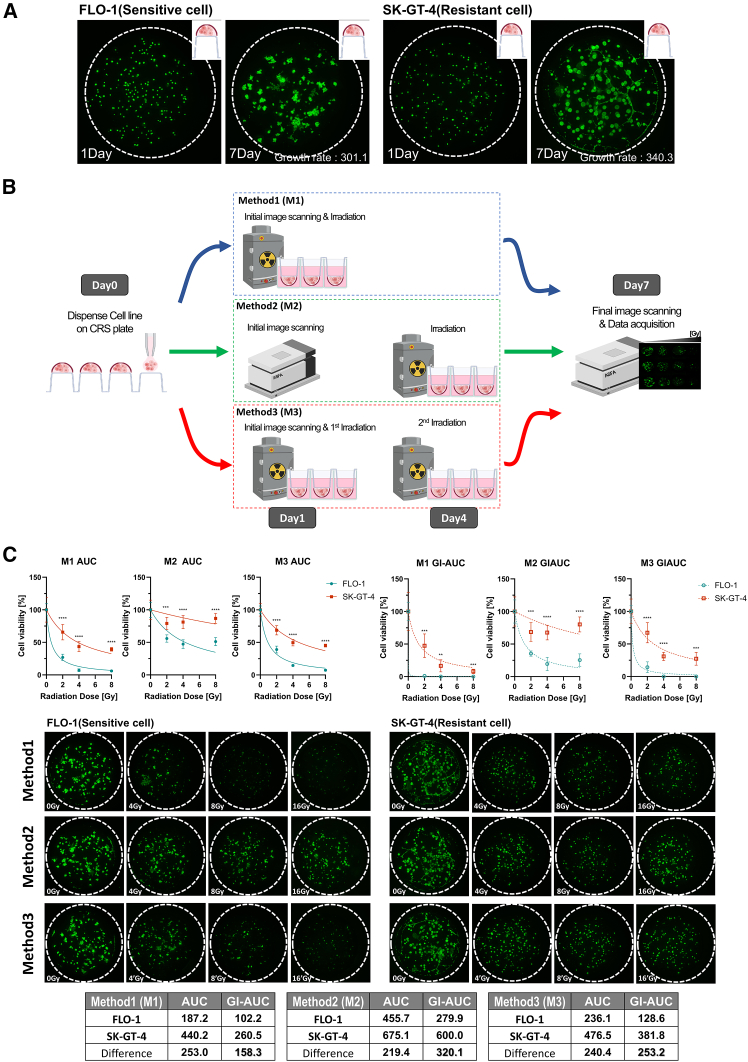
The experimental workflow for radiation sensitivity screening using two esophageal cancer cell lines (A) Growth rates of the two cell lines. (B) Experimental timeline: On day 0, the cells were seeded and cultured on CRS plates. Method 1 (M1): single irradiation on day 1, Method 2 (M2): single irradiation on day 4 and Method 3 (M3): double irradiation on day 1 and day 4. On day 7, cells were stained with a live-cell fluorescent dye (green fluorescence) and imaged to evaluate radiation response. (C) Radiation sensitivity of irradiated FLO-1 and SK-GT-4 cells cultured on CRS plates was quantitatively evaluated by calculating the AUC (area under the cell viability inhibition curve) and GI-AUC (area under the cell growth inhibition curve) values based on the fluorescence imaging results. Data are presented as mean ± SD from *n* = 7 technical replicates. Statistical significance was assessed using one-way ANOVA. ∗∗*p* < 0.01, ∗∗∗*p* < 0.001, and ∗∗∗∗*p* < 0.0001.

Prior to application on the CRS plates (Pillar/Well format), two esophageal cancer cell lines were cultured in BME to assess their growth rates (Figure 1A) and radiosensitivity under three different protocols: single exposure (Method 1 and Method 2) and double exposure (Method 3) (Figure 1B). FLO-1 and SK-GT-4 were identified as radiation-sensitive and radiation-resistant cell lines, respectively. FLO-1 exhibited a strong sensitivity to radiation under all treatment conditions, whereas SK-GT-4 displayed marked resistance.^13^

When comparing the three protocols, statistical and biological analyses revealed that the fractionated M3 protocol provided enhanced analytical resolution and biological amplification compared to the single-dose methods. First, the AUC-DI (Discrimination Index), which indicates the discriminative power of the overall survival curve, was calculated to be 11.48, demonstrating superior discrimination compared to M1 (7.47). Second, the separation window (SW) analysis results showed that M3 recorded a high resolution of 6.57 at low dose (4 Gy), overcoming the limitation of M1 (SW 1.48), which only has discriminative power at high doses. Third, Method 3 maintained stable and reliable Z′-factor values (0.50 at 8 Gy, 0.46 at 16 Gy) in the “excellent to good” range in biological analysis (Table 1). The AUC and GI-AUC were calculated based on cell viability and growth inhibition curves, respectively. Since GI-AUC is calculated by subtracting the day 1 data from the final day data, it specifically captures radiation-associated growth inhibition. The difference in GI-AUC between FLO-1 and SK-GT-4 was greater than that of the conventional AUC, indicating that growth inhibition was more significantly impacted by radiation.

**Table 1:** Statistical analysis results by dose

Method 1 (M1)	FLO1		SK-GT-4		SW	DI	Z′-factor
	Mean	SD	Mean	SD			
4Gy	26.88	4.88	65.60	11.90	1.48	7.47	−0.30
8Gy	7.13	2.19	43.50	7.92	2.42		0.17
16Gy	6.02	1.25	39.25	2.83	10.08		0.63
Method 2 (M2)	FLO1		SK-GT-4		SW	DI	Z′-factor
	Mean	SD	Mean	SD			
4Gy	55.88	5.59	79.06	9.52	1.20	5.26	−0.96
8Gy	47.21	6.72	81.16	10.21	2.30		−0.50
16Gy	51.15	6.23	86.77	7.63	4.12		−0.17
Method 3 (M3)	FLO1		SK-GT-4		SW	DI	Z′-factor
	Mean	SD	Mean	SD			
4Gy	39.04	5.21	76.66	5.56	6.57	11.48	0.14
8Gy	14.50	1.04	53.78	5.48	4.74		0.50
16Gy	7.26	1.26	51.34	6.63	4.22		0.46

As a result, dual irradiation demonstrated improved discrimination between radiation-sensitive and radiation-resistant cell populations. In addition, it has been confirmed that growth rates differ depending on the cell type, and because GI-AUC corrects for initial growth deviations, it may serve as a potentially more informative indicator for personalized assessment. Therefore, Method 3 was selected for subsequent experiments.^14^

### Establishment of patient-derived cancer organoid model on the CRS™ plates

**Figure 2:**
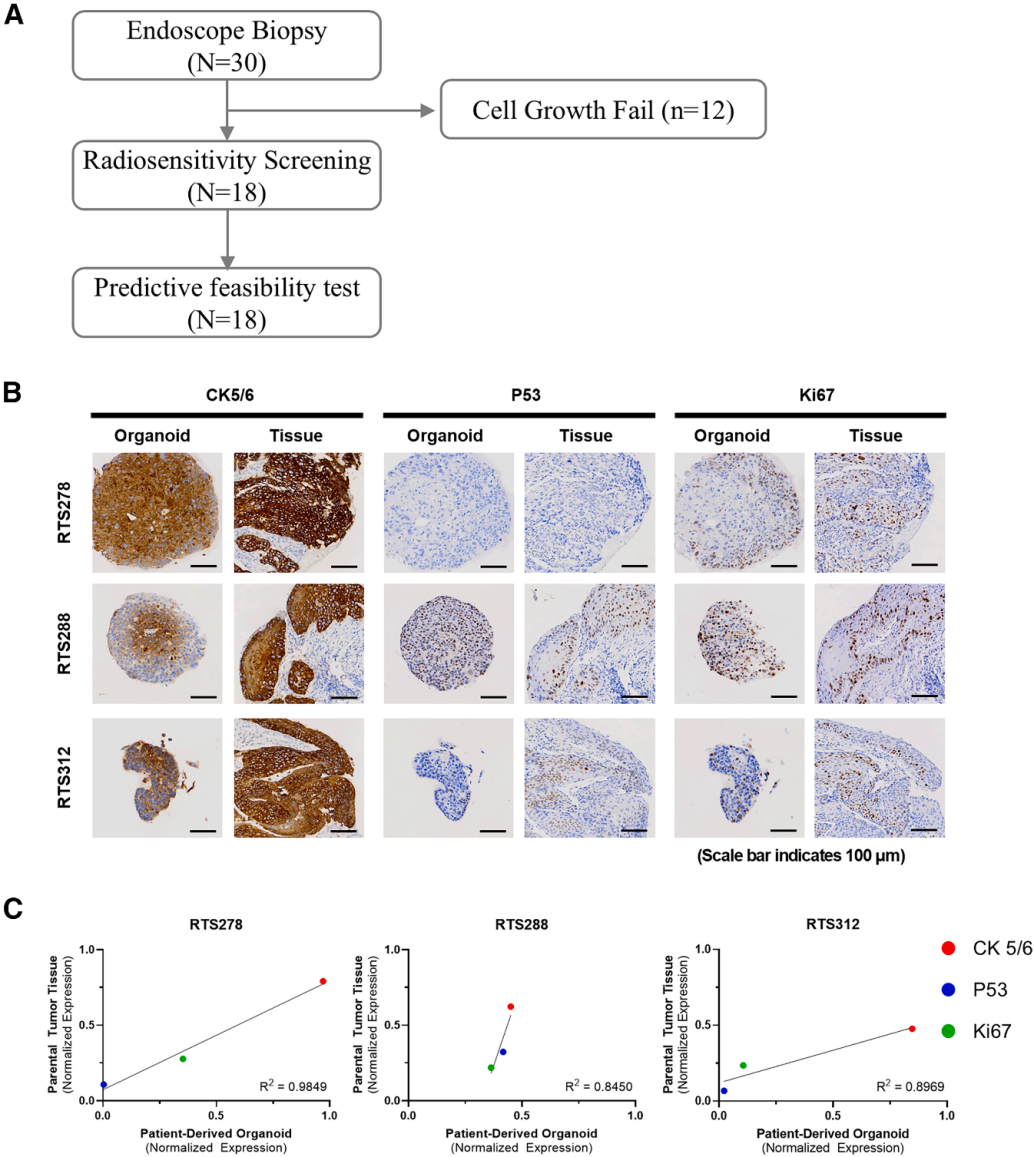
Patient enrollment and histological comparison between organoids and primary tumor tissues (A) Patient enrollment and success rate of organoid culture. Among 30 enrolled patients, organoid formation failed in 12 cases, resulting in a final cohort of 18 cases included in the study. (B) Immunohistochemical analysis of matched primary esophageal tumor tissues and patient-derived esophageal cancer organoids. Samples were stained for CK5/6, p53, and Ki67, and expression patterns were compared between matched tissues and organoids. Scale bars, 100 μm. (C) Quantitative correlation of biomarker expression between parental tissues and PDOs.

Endoscopy was conducted to gather samples from a total of 30 patients with esophageal cancer. Among them, 18 samples were successfully used for radio-response screening (Figure 2A). We could match the clinical data to 18 cases. Information regarding these 18 patients is summarized in Table 2. The IHC of the original tumors was compared with that of matching organoid slides to establish that the PDOs on the CRS™ plates retained attributes similar to the underlying tumors. Only one organoid passage was performed and testing for CK5/6, P53, and Ki67 revealed that the PDOs retained histological features comparable to those of the original tumor (Figures 2B and 2C; Table S1).

**Table 2:** Detailed information of the patients with esophageal cancer

No	ID	Gender	Age	RT dose [mGy]	Pathological response score	Recur	RFS [Month]	cTNM	cTNM score	*Z* score of cTNM	GI-AUC	*Z* score of GI-AUC	Growth rate	*Z* score of growth rate
1	RTS032	M	61	4400	1	0	46	310	3	0.199	800	1.258	86.4	−1.073
2	RTS033	M	60	4400	2	1	5	320	3	0.199	100	−1.109	179.7	−0.014
3	RTS041	M	62	4400	0	1	13	111	5	1.994	0	−1.447	88.0	−1.056
4	RTS053	M	61	4140	0	1	13	310	3	0.199	283	−0.490	141.1	−0.452
5	RTS059	M	61	4400	1	0	44	210	2	−0.698	516	0.298	152.6	−0.322
6	RTS061	M	68	4400	1	0	3	310	3	0.199	259	−0.569	295.1	1.295
7	RTS070	M	69	4140	2	1	12	320	3	0.199	800	1.258	157.7	−0.264
8	RTS130	M	59	4140	0	1	13	1b10	1	−1.595	800	1.258	123.8	−0.649
9	RTS163	F	48	4140	0	0	39	310	3	0.199	0	−1.447	73.9	−1.215
10	RTS205	M	43	4140	3	1	7	310	3	0.199	800	1.258	310.9	1.475
11	RTS221	M	64	4140	0	1	7	210	2	−0.698	347	−0.274	153.3	−0.314
12	RTS222	M	48	4140	3	1	6	320	3	0.199	666	0.807	168.0	−0.148
13	RTS229	M	62	4140	0	0	26	1b10	1	−1.595	168	−0.879	151.4	−0.336
14	RTS232	M	70	4140	2	1	12	310	3	0.199	561	0.450	237.1	0.637
15	RTS249	M	62	4140	1	1	7	321	5	1.994	126	−1.019	418.6	2.696
16	RTS250	F	68	4140	2	1	1	310	3	0.199	275	−0.516	216.2	0.400
17	RTS290	M	64	4140	0	0	33	1b10	1	−1.595	800	1.258	116.8	−0.728
18	RTS345	M	61	4140	0	0	27	320	3	0.199	399	−0.096	187.1	0.069

RFS, recurrence-free survival (months).

### Radiation sensitivity screening using CRS™ plate

**Figure 3:**
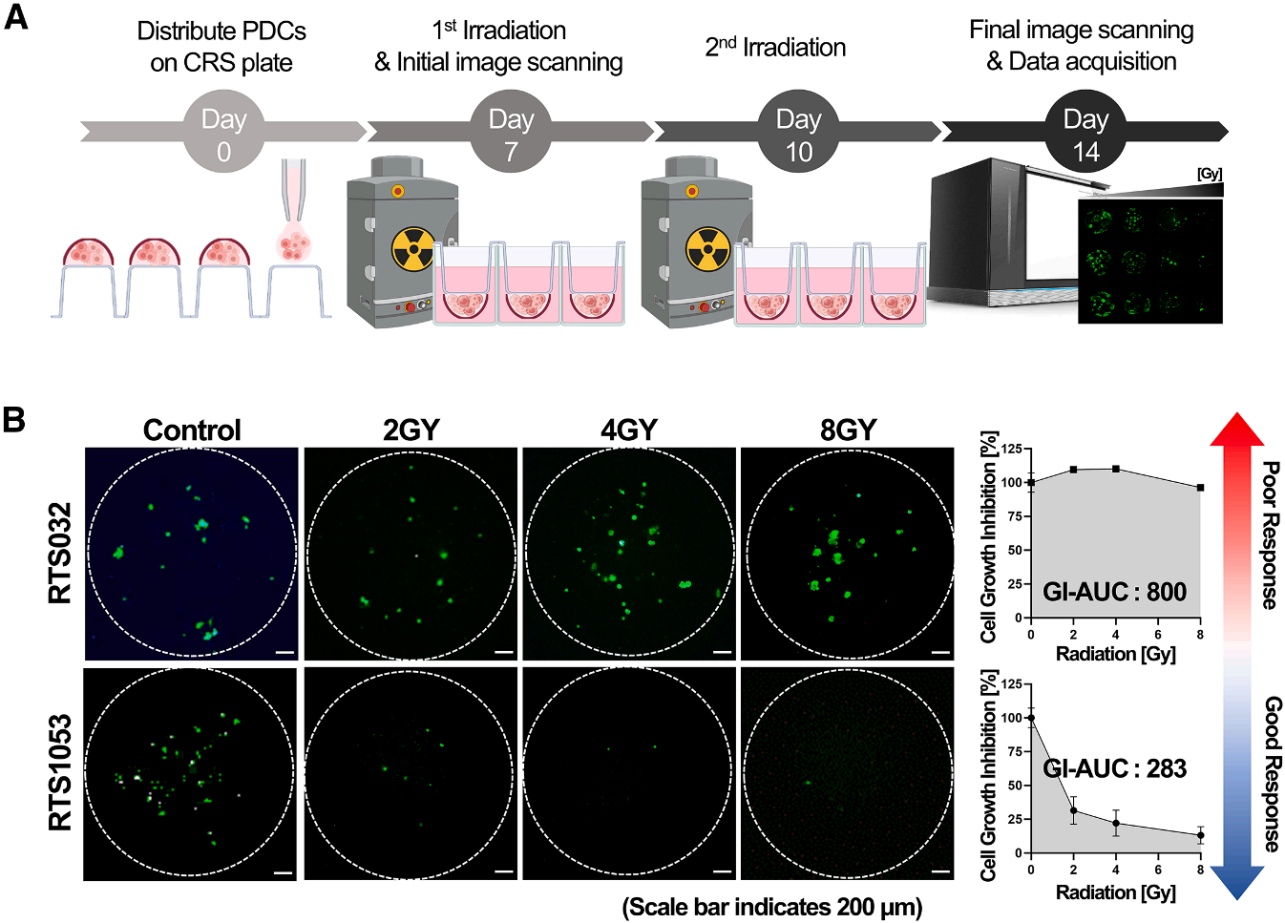
Experimental workflow for radiation sensitivity screening using patient-derived esophageal cancer organoids (A) Experimental Timeline: On day 0, organoids were seeded and cultured. On day 7, the first radiation treatment was administered, followed by a second treatment on day 10. On day 14, the organoids were stained with a live-cell fluorescent dye (green fluorescence) and imaged to assess their radiation response. (B) Radiation sensitivity of irradiated patient-derived esophageal cancer organoids was quantitatively evaluated by calculating the GI-AUC based on the fluorescence imaging results. Data are presented as mean ± SD from *n* = 3 technical replicates. Scale bars, 200 μm.

Specimens obtained through endoscopy at the medical center were dissociated into single cells and seeded onto CRS™ plates in a three-dimensional culture system to develop a radiation response screening model. The CRS™ plate, consisting of 384 pillars, was compatible with standard 384-well plates (Figures S1A–S1D). We have previously reported the development of this platform for predicting radiation response.^10^ Based on the fractionated irradiation strategy optimized in the cell-line experiments, the irradiation timing was adapted for PDO experiments to day 7 and day 10 to allow sufficient 3D organoid formation and stabilization before irradiation. Following a 7-day stabilization period, primary irradiation was performed on all 18 PDOs. Cell staining was performed on the control organoids to calculate the growth rate. Irradiation was conducted at the same dose after three days, and cell viability was validated on the 14th day to determine the GI-AUC and growth rate. Three-dimensional tumor models generated on CRS™ plates were treated with radiation doses of 0, 2, 4, and 8 Gy. On day 7 (before the first irradiation) and day 14, live tumor cells were stained with Calcein AM, and mean colony areas were calculated using automated image analysis software (ASFA Scanner ST, Medical & Bio Device) (Figure 3A). Growth rates were determined based on the increase in mean colony size. Growth inhibition at each radiation dose was calculated from the final images acquired on day 14 to generate radiation dose-response curves. The GI-AUC values were automatically calculated from the radiation dose-response curves using GraphPad Prism 10 (Figure 3B). The maximum GI-AUC was 800, and the case where the AUC exceeded 800 was considered the maximum value because it was higher than the value normalized by the control. Table 2 shows the GI-AUC values and growth rates of cells from 18 patients. The average growth rate and GI-AUC of the 18 patients were 178 and 427, respectively. For comparing the two parameters, the growth rates and GI-AUC values were standardized using the mean and standard deviation to obtain Z-scores.

### Correlation between the cancer organoid-based diagnosis and reactivity prediction indices and the clinical response of patients

**Figure 4:**
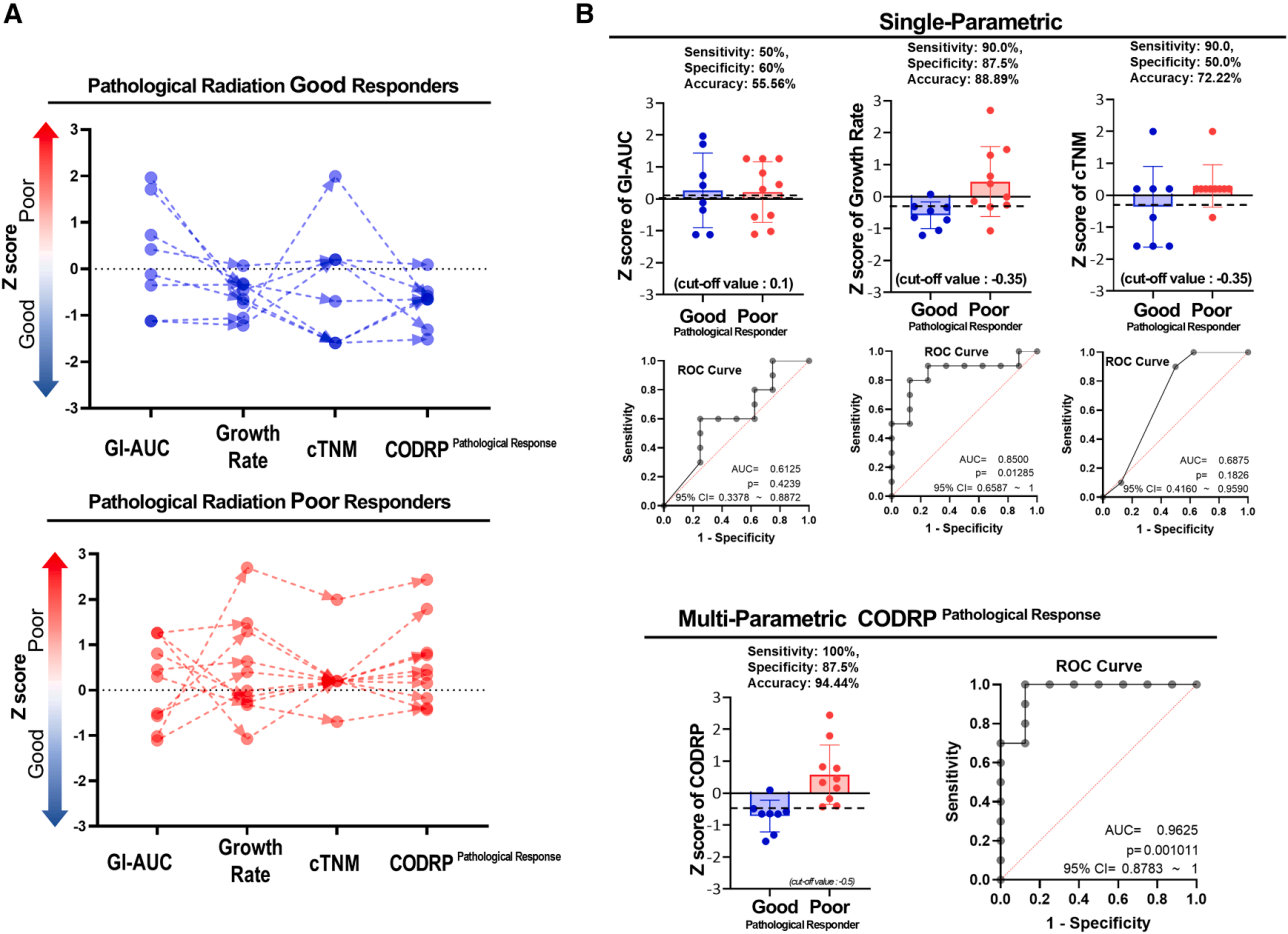
Quantitative analysis of radiation sensitivity testing for predicting the pathological response to neoadjuvant chemoradiotherapy (A) Z-scores of the GI-AUC, growth rate, cTNM, and CODRP were compared between pathological radiation good responders (left, blue) and poor responders (right, red). In the good responder group (*n* = 8), growth rates and CODRP values exhibited a consistently low range, indicating a good correlation with clinical outcomes, whereas GI-AUC and cTNM showed wide variability. In the poor responder group (*n* = 10), the GI-AUC, growth rate, and cTNM values displayed broader variability, whereas the CODRP values remained relatively stable and correlated well with clinical outcomes. Dotted lines connect individual patient data points across parameters. (B) Comparison of the predictive performance of single- and multi-parameter models for assessing radiation sensitivity. The sensitivity and specificity for each single-parameter model were as follows: GI-AUC (50%, 60%), growth rate (87.5%, 90.0%), and cTNM staging (50.0%, 90.0%). The multi-parameter pathological RT response-predicting CODRP model demonstrated superior performance with sensitivity and specificity at 87.5% and 100.0%, respectively. Patients were stratified into good and poor responders using optimized cutoff values that maximized both the sensitivity and specificity. Data are represented as mean ± SD.

Considering the variability in patient responses to NCRT, we evaluated the Z-scores of GI-AUC, growth rate, cTNM stage, and CODRP ^Pathological response^ for each of the 18 patients. We then compared the pathological response to NCRT with each individual parameter and with the multiparameter index, CODRP ^Pathological response^. In Figure 4A, the predictive performances of GI-AUC, growth rate, cTNM, and CODRP ^Pathological response^ were compared based on the pathological response to NCRT. In the good pathological responder group (*n* = 8), growth rate and CODRP ^Pathological response^ index values were consistently low, demonstrating a strong correlation with the clinical outcomes. In contrast, GI-AUC and cTNM values showed wide variability, suggesting a weaker correlation with the pathological response. Similarly, in the poor responder group (*n* = 10), the GI-AUC, growth rate, and cTNM values were broadly distributed, whereas the CODRP ^Pathological response^ index remained consistently high and was better aligned with the clinical outcomes. These findings suggest that the CODRP ^Pathological response^ index, which incorporates growth rate as a key parameter, reflects patient responses to NCRT more accurately than do individual markers such as GI-AUC or cTNM.

As shown in Figure 4B, the Z-scores of the individual parameters and CODRP ^Pathological response^ were compared between the good and poor pathological responder groups. For GI-AUC, the maximum sensitivity and specificity were 50% and 60%, respectively, with the average *Z* score being slightly higher in poor responders than in good responders. The maximum sensitivity and specificity for the cell growth rate were 87.5% and 90%, respectively, demonstrating the best predictive performance among the three single parameters. We subsequently developed a multi-parameter model of the CODRP ^Pathological response^, integrating clinical cancer stage, PDO growth rate, and GI-AUC. The CODRP ^Pathological response^ showed significantly improved sensitivity (87.5%) and specificity (100%). The area under the ROC curve (AUC) for the CODRP ^Pathological response^ was 0.9625, indicating that the CODRP ^Pathological response^ showed strong predictive performance for pathological response.

**Figure 5:**
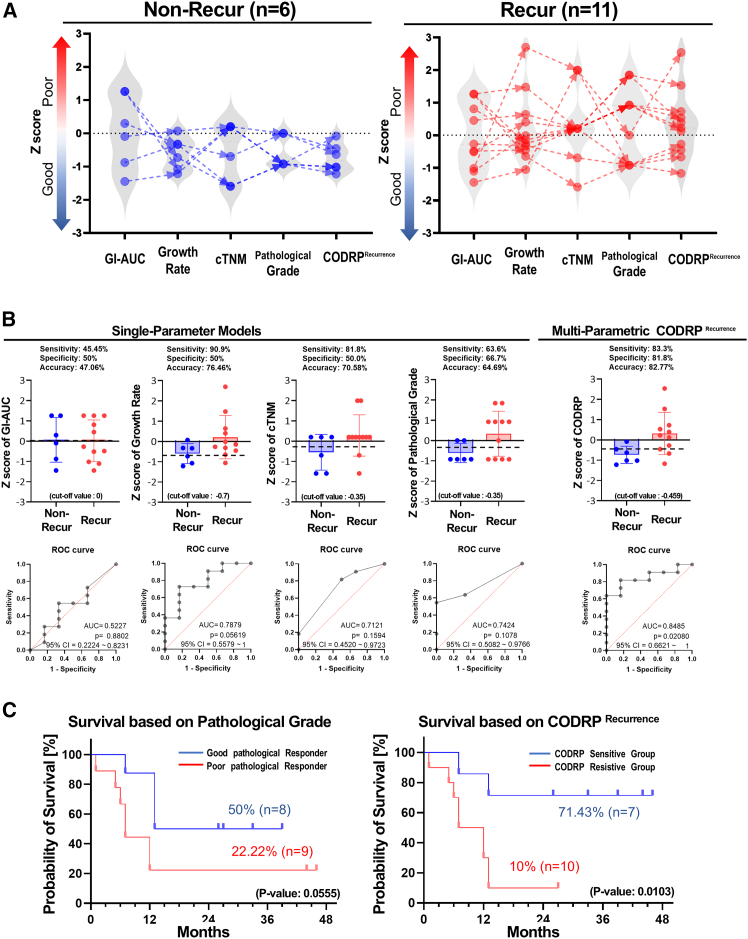
Quantitative analysis of radiation sensitivity testing for predicting recurrence risk (A) Z-scores of the GI-AUC, growth rate, cTNM, pathological grade, and CODRP were compared between non-recurrence group (left, blue) and recurrence group (right, red). In the non-recurrence group (*n* = 6), growth rates, pathological grade, and CODRP values exhibited a consistently low range, indicating good correlation with clinical outcomes, whereas GI-AUC and cTNM showed wide variability. In the recurrence group (*n* = 11), GI-AUC, growth rate, pathological grade, and cTNM values displayed broader variability, whereas the CODRP values remained relatively stable and correlated well with clinical outcomes. Dotted lines connect individual patient data points across parameters. (B) Comparison of the predictive performance of single- and multi-parameter models for models for predicting recurrence risk. The sensitivity and specificity for each single-parameter model were as follows: GI-AUC (50.0%, 54.5%), growth rate (50%, 90.9%), and cTNM staging (50.0%, 81.8%). The sensitivity and specificity for each pathological grade were 66.7% and 63.6%, respectively. The multiparameter recurrence-predicting CODRP model demonstrated superior performance with sensitivity and specificity of 83.3% and 81.8%, respectively. Patients were stratified into good and poor responders using optimized cutoff values that maximized both the sensitivity and specificity. Data are represented as mean ± SD. (C) Kaplan–Meier survival analysis for recurrence prediction comparing single-parameter and multi-parameter models. The CODRP model (*p* = 0.0103) showed a significantly better ability to distinguish between recurrence groups compared with that of the single-parameter pathological grade model (*p* = 0.0555).

As shown in Figure 5, the CODRP ^Recurrence^, which incorporates four parameters (GI-AUC, growth rate, cTNM stage, and pathological grade), was used to predict recurrence risk. CODRP ^Recurrence^ exhibited significantly higher accuracy in predicting recurrence compared to that of single-parameter models and pathological grade alone. Figure 5A shows a comparison of the predictive performances of GI-AUC, growth rate, cTNM stage, pathological grade, and CODRP ^Recurrence^ based on recurrence after surgery. Pathological grade was assessed postoperatively in patients who underwent NCRT. In the non-recurrence group (*n* = 6), the growth rates, cTNM stages, pathological grades, and CODRP ^Recurrence^ index values were consistently low, showing a strong correlation with favorable clinical outcomes. In contrast, the GI-AUC values showed wide variability, suggesting a weaker correlation with recurrence. In the recurrence group (*n* = 11), the GI-AUC, growth rate, and cTNM values were broadly distributed, whereas the pathological grade and CODRP ^Recurrence^ index values remained consistently high and showed a stronger alignment with clinical outcomes. These findings suggest that the CODRP ^Recurrence^ index, which integrates four parameters, reflects the recurrence risk after NCRT more accurately than individual markers such as pathological grade (Figure 5B). The AUC of the ROC curve for CODRP ^Recurrence^ was 0.8485, indicating strong predictive accuracy for recurrence after surgery.

RFS was further evaluated using Kaplan–Meier analysis with the log-rank test (Figure 5C). Although the pathological response grade is conventionally considered an important prognostic factor, our study did not show a statistically significant difference in RFS between the good and poor pathological responder groups (2-year RFS: 50.0% vs. 22.2%, *p* = 0.056). In contrast, prognostic discrimination was significantly improved when survival analysis was performed using the CODRP ^Recurrence^ index, a multiparameter model integrating GI-AUC, PDO growth rate, cTNM stage, and pathological grade. Based on this model, patients classified as sensitive according to the CODRP ^Recurrence^ index showed significantly better outcomes than those classified as resistant (2-year RFS: 71.4% vs. 10.0%, *p* = 0.010).

**Figure 6:**
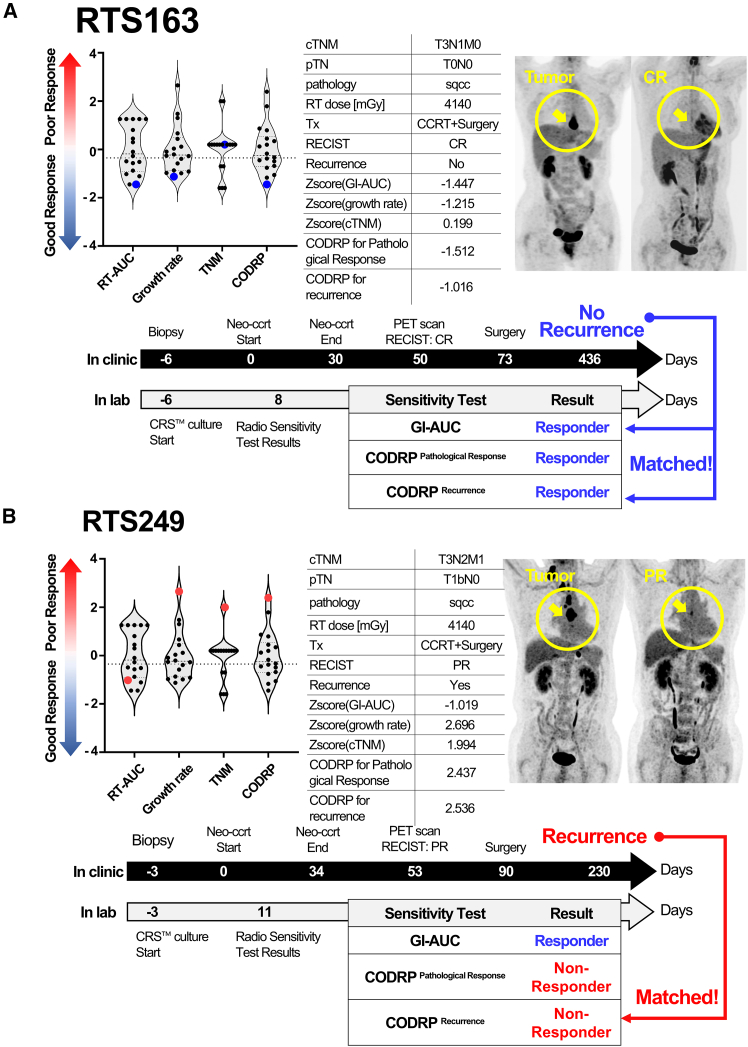
Correlation between radiation sensitivity test results and recurrence in patients with esophageal cancer (A) Patient RTS163 underwent chemoradiotherapy followed by surgery, achieving a clinical complete response and experienced no recurrence during follow-up. The CODRP indices for pathological response and recurrence predicted the patient as a responder in the radiation sensitivity test. Both the CODRP indices and GI-AUC index were consistent with the observed clinical outcome. (B) Patient RTS249 received the same treatment but exhibited a clinical partial response and later developed recurrence at 230 days. The CODRP indices predicted the patient as a non-responder, which matched with the clinical outcome. However, the GI-AUC index (*Z* score = −1.019) predicted the patient as a responder. The CODRP indices were were more consistent with the clinical course than the GI-AUC index in this case.

Figure 6 shows two examples. Patient RTS163 did not experience recurrence after NCRT followed by surgery, whereas RTS249 did. The details of the clinical response, pathological response, and parameter values are presented in Figure 6. The CODRP index correlated well with the clinical course.

## Discussion

In this study, we developed a predictive model for pathologic treatment response in esophageal SCC after NCRT by utilizing organoids derived from pretreatment diagnostic biopsy samples. We also predicted recurrence-free survival based on organoid radiation response, growth, cTNM stage, and pathological response. Due to the limited size of clinical biopsy samples, performing multiple technical replicates for all PDOs was not feasible without introducing additional passages, which could alter tumor characteristics, representing a limitation of this study. However, the robustness of the assay is supported by multiple quality metrics, including a high SW, and a strong DI, all indicating excellent assay performance. Radiation sensitivity data were successfully obtained within two weeks. A limitation of this study is the relatively low organoid establishment rate (18/30, 60%). This may be attributed to factors such as limited biopsy size, low tumor cellularity, or the presence of necrotic tissue, which can reduce the viability of cells required for organoid formation. To improve clinical applicability, further optimization of tissue acquisition, processing protocols, and culture conditions will be necessary to increase organoid establishment efficiency in future studies. Compared with the single-parameter model using GI-AUC (conventional model), the multi-parameter CODRP ^Pathologic response^ model, which integrates GI-AUC, growth rate, and cTNM, significantly improved the predictive performance (sensitivity of 87.5% and specificity of 100%). Notably, in the CODRP ^Pathologic response^ sensitive group, 87.5% of patients successfully achieved pCR, which was significantly higher than the 12.5% pCR rate observed in the CODRP ^Pathologic response^ resistive group. Furthermore, the survival outcomes were significantly better in the CODRP ^Recurrence^ sensitive group than in the CODRP ^Recurrence^ resistive group, highlighting the clinical utility of this predictive approach. The findings of this study underscore the remarkable heterogeneity observed in the radiation response of PDO models, which is a critical consideration in the development of effective personalized treatment strategies. This variability can be attributed to inherent differences among individual patients, including their specific cancer stage progression, cellular proliferation rates, and other intrinsic tumor characteristics. Our results highlight the limitations of relying solely on single-parameter models such as GI-AUC to accurately predict radiotherapy outcomes. GI-AUC alone yielded a sensitivity of only 50%, emphasizing the need for integrating multiple factors to comprehensively capture the individuality of treatment responses.

Previous CODRP work applied a multiparametric organoid-based framework to anticancer drug sensitivity prediction in ALK-rearranged non-small cell lung cancer.^12^ In contrast, the present study extends this framework to radiation sensitivity assessment and NCRT response prediction in esophageal SCC. To address this challenge, we applied the CODRP multiparameter model that synergistically combines clinical cancer stage, PDO growth rate, and RTauc values. Remarkably, this approach demonstrated a significantly improved predictive performance compared to that of single-parameter models, achieving 87.5% sensitivity and 100% specificity. This substantial enhancement in accuracy underscores the value of adopting a multifactorial approach accounting for the diverse biological and clinical factors influencing radiation response. Furthermore, by incorporating the pathological response grade score as an additional parameter, the CODRP ^Recurrence^ model exhibited superior accuracy in predicting tumor recurrence compared to that of models utilizing the pathological response grade alone. These findings were further supported by the survival analysis.

Our organoid-based radiation sensitivity tests provided results just two weeks after biopsy acquisition, allowing for the pretreatment prediction of concurrent chemoradiotherapy (CCRT) response. Conventionally, the treatment response is assessed after the completion of CCRT, either through post-treatment imaging or surgical pathology. However, by enabling early response prediction before treatment initiation, our model facilitates a timely prognostic estimation, allowing both patients and clinicians to make informed decisions regarding subsequent treatment strategies. Given that treatment response is a well-established prognostic factor,^15^ early identification of radiosensitivity naturally leads to faster prognostic assessment and improved clinical decision-making. The ability to predict the CCRT response before treatment initiation has significant implications beyond early prognoses. For patients expected to respond well to CCRT, a non-operative management strategy, such as “watch and wait” after achieving clinical CR, can be considered, as currently being investigated in the Surgery As Needed for Esophageal Cancer (SANO) trial.^16^ Additionally, in patients at risk of radiation-induced toxicity, dose reduction may be a feasible strategy for minimizing adverse effects while maintaining therapeutic efficacy. Conversely, for patients predicted to be radioresistant, early surgical intervention or treatment intensification should be prioritized to improve clinical outcomes. Moreover, in cases where definitive CCRT is being considered, either to preserve organ function or for patients unable to tolerate surgery, pretreatment response prediction can aid in refining treatment decisions and optimizing therapeutic planning. However, the clinical utility of this predictive model ultimately depends on its accuracy. We demonstrated that by employing a multi-parameter model integrating GI-AUC, growth rate, and clinical staging, we could achieve a more accurate prediction of pCR than that with single-parameter models.

To validate the CODRP ^Recurrence^ model, a cohort of 17 patients (11 with recurrence and 6 without recurrence) was used; however, the limited availability of clinical specimens posed challenges to establishing a stable model. Therefore, internal validation using bootstrap resampling was performed to assess the stability and potential optimism of the recurrence prediction model. The apparent performance of the standard multivariable logistic regression model, which included GI-AUC, Growth Rate, cTNM, and pathologic response, was first evaluated using the original dataset. Because of the small sample size and instability caused by separation in some bootstrap resamples, penalized logistic regression was additionally used during the bootstrap procedure to obtain more stable internal validation estimates. A total of 2,000 bootstrap resamples were generated, and model performance was assessed using the area under the receiver operating characteristic curve (AUC), accuracy, sensitivity, specificity, and Brier score. Optimism-corrected performance was calculated by subtracting the average bootstrap optimism from the apparent performance. Calibration was assessed using the calibration intercept and slope.

In the original dataset, the standard multivariable logistic regression model showed an apparent AUC of 0.8485. However, given the small sample size and instability during resampling, bootstrap-based internal validation was performed using penalized logistic regression. The penalized model showed an apparent AUC of 0.833, which decreased to an optimism-corrected AUC of 0.735 after bootstrap validation. At a probability cutoff of 0.5, the optimism-corrected accuracy, sensitivity, and specificity were 0.585, 0.744, and 0.370, respectively. The Brier score increased from 0.158 to 0.222 after optimism correction. These findings suggest that the model has moderate discriminatory ability, but may be affected by overfitting, particularly given the limited sample size. Calibration analysis showed an apparent intercept of 0.132 and a slope of 1.531; however, these estimates were unstable across bootstrap resamples.

In summary, a multiparameter predictive model for pathologic treatment response in esophageal SCC following NCRT was evaluated in this study using patient-derived cancer organoids. The CODRP model, which combines GI-AUC, PDO growth rate, and clinical staging, showed higher apparent predictive performance than single-parameter models (sensitivity, 87.5%; specificity, 100%). Additionally, the proposed CODRP model showed potential for stratifying post-treatment survival outcomes compared to that of single-parameter models using only pathological grades. Early prediction of treatment responses may support future personalized treatment strategies and clinical decision-making, pending validation in larger independent cohorts. Collectively, these findings suggest that the CODRP model may help guide more precise treatment strategies for patients with esophageal SCC, but further validation in larger independent cohorts is required before clinical implementation.

### Limitations of the study

This study demonstrated, in a small patient cohort, that the CODRP model predicts radiotherapy response based not only on the radiation sensitivity of PDOs but also on organoid growth rate and the patient’s clinical tumor stage. However, to further refine the predictive accuracy of the model, improve its generalizability, and establish its clinical utility in guiding personalized treatment strategies for esophageal cancer, independent external validation in larger patient cohorts through clinical trials is required. The current findings should therefore be interpreted as feasibility-level evidence rather than as a fully validated clinically deployable assay.

For clinical implementation of the radiotherapy sensitivity assay, it is necessary to demonstrate that, among patients classified as radiation-responsive, reducing radiation dose can decrease treatment-related toxicity without compromising therapeutic efficacy. In addition, for radiation-resistant patients, clinical studies are needed to verify whether earlier surgical intervention and adjuvant chemotherapy can improve outcomes compared with current standard treatment approaches.

Although a total of 30 endoscopic biopsy samples were obtained from patients with esophageal cancer, 12 were excluded from the analysis because of low cell growth rates. Review of these excluded samples revealed no clear association with age or cTNM stage. However, samples with low initial cell viability tended to show poorer culture outcomes. Although cells were seeded uniformly according to the number of viable cells, 6 of the 12 failed samples exhibited less than 50% viability at the time of cell isolation. Notably, approximately 3 cases in the high-growth group also showed viability below 50%, and therefore no statistically significant difference was identified. While the limited sample size precluded definitive statistical conclusions, these findings suggest that initial cell viability may be an important factor influencing the success of organoid culture and should be further investigated in future organoid-based studies, together with optimization of biopsy processing, culture medium composition, and ECM conditions.

Given the limited amount of biopsy-derived material and the need to preserve early-passage PDO characteristics, formal intra-assay reproducibility testing using additional technical replicates was not performed for PDOs. Although raw QuPath-derived CK5/6 H-scores and p53/Ki-67 positive-cell percentages are provided for the available matched tumor-PDO pairs, tumor-organoid fidelity was assessed using a limited number of markers and cases. Broader genomic or transcriptomic concordance between parental tumors and PDOs was not evaluated and should be addressed in future studies.

## Resource availability

### Lead contact

Requests for further information and resources should be directed to and will be fulfilled by the lead contact, Dong Woo Lee (dw2010.lee@gmail.com).

### Materials availability

This study did not generate new unique reagents. Patient-derived cells and organoids generated from clinical biopsy specimens are not publicly available because of restrictions related to patient consent and institutional ethical approval. Further information regarding materials used in this study is available from the lead contact upon reasonable request.

### Data and code availability

• All data reported in this paper are included in the main text and supplemental information. Additional de-identified data supporting the findings of this study will be shared by the lead contact upon reasonable request, subject to institutional and ethical approvals.
• This paper does not report original code. The equations used to calculate the CODRP indices are provided in the STAR Methods.
• Any additional information required to reanalyze the data reported in this paper is available from the lead contact upon request.

## Acknowledgments

This research was supported by the Bio & Medical Technology Development Program of the National Research Foundation (NRF) funded by the Korean government (MSIT) (grant no. RS-2023-00222910). This research was supported by a grant no. RS-2024-00332142 from Ministry of Food and Drug Safety. This work was supported by the Korea Medical Device Development Fund grant funded by the Korean government (the Ministry of Science and ICT; the Ministry of Trade, Industry and Energy; the Ministry of Health & Welfare; and the Ministry of Food and Drug Safety) (project no. RS_2023-00255627). This research was supported by a grant of the Korean ARPA-H Project through the Korea Health Industry Development Institute (KHIDI), funded by the Ministry of Health & Welfare, Republic of Korea (grant no. RS-2024-00512449). This work was supported by the National Research Foundation of Korea (NRF) grant funded by the Korean government (MSIT) (grant no. RS-2023-00212410). This work was supported by Chungnam National University. This work was supported by the National Research Foundation of Korea (NRF) grant funded by the Korean government (the Ministry of Science and ICT) (grant no. NRF-2020M2D9A3094087).

## Author contributions

Conceptualization, D.W.L. and D.O.; methodology, E.J., Y.C., and B.K.; validation, E.J., J.Y.B., and S.Y.L.; formal analysis, E.J., J.Y.B., S.Y.L., D.B.P., M.K.C., D.W.L., and D.O.; writing – original draft, E.J., S.Y.L., and D.W.L.; writing – review and editing, E.J., J.Y.B., S.Y.L., D.B.P., M.K.C., D.W.L., and D.O.; supervision, D.W.L. and D.O.; project administration, D.W.L. and D.O. All authors reviewed and approved the final manuscript.

## Declaration of interests

The authors declare no competing interests.

## STAR★Methods

### Key resources table

**Table:** 

REAGENT or RESOURCE	SOURCE	IDENTIFIER
**Antibodies**		

Anti-CK5/6 antibody	Abcam	Cat# ab17133
Anti-P53 antibody	Abcam	Cat# ab32049; RRID: AB_776982
Anti-Ki67 antibody	Abcam	Cat# ab16667; RRID: AB_302459

**Biological samples**		

Endoscopic biopsy specimens from patients with esophageal cancer	Patients with esophageal cancer	SMC IRB file number 2020-09-002-007

**Chemicals, peptides, and recombinant proteins**		

Dulbecco’s Modified Eagle Medium	Gibco	Cat# 11965092
RPMI-1640 medium	Gibco	Cat# 11875093
Fetal bovine serum	Gibco	Cat# A5670701
Penicillin-streptomycin	Gibco	Cat# 15070063
Trypsin 0.25% solution	Gibco	Cat# 25200056
Basement membrane extract type 2	R&D Systems	Cat# 3536-005-02
Calcein AM	Invitrogen	Cat# C3099
DNase I	Sigma-Aldrich	Cat# 10104159001
Collagenase/hyaluronidase	STEMCELL Technologies	Cat# 07912
Amphotericin B	Invitrogen	Cat# 15290018
Trypan blue	Gibco	Cat# 15250061
R-spondin 1	PeproTech	Cat# 120–38
Noggin	PeproTech	Cat# 120-10C
Y-27632	AdooQ	Cat# A11001
B-27 supplement	Gibco	Cat# 17504044
N-acetyl-L-cysteine	Sigma-Aldrich	Cat# A9165
Leu15-gastrin I	Sigma-Aldrich	Cat# G9145
Recombinant human insulin-like growth factor 1	Welgene	Cat# LS-038-01
Recombinant human fibroblast growth factor 2	PeproTech	Cat# 100-18C
Afamin/Wnt3a conditioned medium	MBI	Cat# J2001

**Experimental models: Cell lines**		

FLO-1	ECACC	Cat#11012001
SK-GT-4	ECACC	Cat#11012007

**Software and algorithms**		

GraphPad Prism 10	GraphPad Software	Prism 10
QuPath	QuPath	v0.6.0
ASFA image analysis software	Medical & Bio Device	v2.1

**Other**		

CRS™ plate	Medical & Bio Decision	custom-designed
ASFA™ Spotter DN	Medical & Bio Decision	ASFA™ Spotter DN
ASFA™ Scanner ST	Medical & Bio Decision	ASFA™ Scanner ST
CellRad irradiator	Precision X-ray, Inc.	CellRad+
Bond-III stainer	Leica Microsystems	Bond-III
Ocus 40 digital microscope scanner	Grundium Oy	Ocus 40

### Experimental model and study participant details

#### Human participants

The acquisition of tissues from patients with esophageal cancer and the relevant experimental protocols were approved by the Institutional Review Board of Samsung Medical Center (SMC IRB file number 2020-09-002-007), and informed consent was obtained from all individuals. This study was conducted in accordance with all the relevant ethical regulations and guidelines for human specimen research. Detailed information on the patients with esophageal cancer is presented in Table 2; Figure S2 describes the detailed procedure for isolating patient-derived cells from esophageal cancer tissues.

In total, 30 patients with esophageal cancer were enrolled through endoscopy. The failure rate on the CRS™ plates was 40% with 12 cases showing no organoid growth. 18 samples were successfully used for radio-response screening. We could match the clinical data to 18 cases (Figure 2A). For recurrence analysis, one patient who was lost to immediate follow-up was excluded, and the analysis was conducted with the remaining 17 patients. Age and sex information for the analyzed patients is presented in Table 2. The influence of sex or gender on the results could not be evaluated because of the limited sample size and male-predominant cohort.

#### Esophageal cell line culture

FLO-1 cells were grown in Dulbecco’s Modified Eagle Medium (DMEM) (Gibco) with 10% fetal bovine serum (FBS) and penicillin/streptomycin (Gibco). SK-GT-4 cells were grown in Roswell Park Memorial Institute (RPMI)-1640 (Gibco), which was supplemented with identical additives as DMEM. All cells were passaged using 0.25% trypsin solution (Gibco) and kept in a 37°C incubator with 5% CO2. 70% basement membrane extract type 2 (BME, 3536-005-02, R&D Systems) was added to all cell lines in the 3D culture.

### Method details

#### Cancer cell isolation from patients

The acquisition of tissues from patients with esophageal cancer and the relevant experimental protocols were approved by the Institutional Review Board of Samsung Medical Center (SMC IRB file number 2020-09-002-007), and informed consent was obtained from all individuals. This study was conducted in accordance with all the relevant ethical regulations and guidelines for human specimen research. Detailed information on the patients with esophageal cancer is presented in Table 2. Endoscopic biopsy samples from patients with esophageal squamous cell carcinoma were enzymatically digested in Advanced DMEM/F12 (Gibco) containing 200 U/mL DNase I (Sigma-Aldrich), 10% collagenase/hyaluronidase (STEMCELL Technologies), 0.2% amphotericin B (Invitrogen), and 1% penicillin-streptomycin. After a 1-h incubation at 37°C in a shaking incubator, dissociated cells were filtered through a 70 μm strainer (SPL) and centrifuged at 500 RCF for 3 min. Cell viability and concentration were determined using trypan blue staining. After viability assessment, dissociated cells were used for 3D organoid culture on CRS plates as described below. Hereafter, dissociated cells isolated from patient biopsy specimens are referred to as patient-derived cells (PDCs), and PDC cultures are referred to as patient-derived organoids (PDOs) once they form stable 3D structures during the culture period.

#### Organoid formation on CRS™ plate

On a CRS™ plate (Pillar/well culture plate), a 1.5 μL mixture of patient-derived cells (PDCs) and basement membrane extract type 2 (BME, #3536-005-02, R&D systems) was distributed manually. To prevent early BME gelation, the pillar plate was positioned onto the blank 384-well plate and placed at 4°C. After 20 min, the PDC-containing BME was gelled for 1 h at 37°C. To cultivate PDCs as patient-derived organoids (PDOs), we cultured them in basal medium with 1 μg/mL R-spondin1 (#120-38, PeproTech), 100 ng/mL Noggin (120-10C, PeproTech), 10 μM Y-27632 (#A11001, AdooQ), 1× B-27 supplement (#17504044, Gibco), 1.25 mM N-acetyl-L-cysteine (#A9165, Sigma Aldrich), 10 nM Leu15-Gastrin I (#G9145, Sigma Aldrich), 100 ng/mL recombinant human Insulin-like Growth Factor (IGF)-1 (#LS-038-01, Welgene), 50 ng/mL recombinant human Fibroblast Growth Factor (FGF)-2 (#100-18C, PeproTech), and 10% Afamin/Wnt3a CM (#J2001, MBI).

#### Experimental workflow of radiation sensitivity test

As shown in Figures 1A and 3B, esophageal cancer cell lines and patient-derived cells (PDCs) were cultured in BME gel to assess their growth rates and radiosensitivity. The pillar-based system ensures uniform ECM microenvironments, which is essential for reproducible radiation response assessment. We have previously reported a platform for 3D culture and high-throughput screening (HTS) of patient-derived organoids (PDOs) using a disposable nozzle-based automated cell spotter (ASFA Spotter DN, Medical & Bio Device), which enables precise and reproducible dispensing of cell–ECM mixtures.^12^^,^^17^^,^^18^ While these models are initially seeded as dissociated cells, they are referred to as patient-derived organoids (PDOs) once they form stable 3D structures during the culture period. The two cell lines formed colonies at a concentration of 200 cells/1.5 μL in BME, and the growth rates were calculated by dividing the mean colony area at 7 days by the mean cell area at 1 day as shown in Figure 1B. For PDCs, colonies were formed at a concentration of 10^4^ cells/1.5 μL, and growth rate was calculated by dividing the mean colony area at 14 days by the mean colony area at 7 days. Esophageal cell lines and PDOs were exposed to the specified dosage of radiation (2, 4, and 8 Gy) using the CellRad (Precision X-ray, Inc.). To assess the effects of radiation, the viability of cells exposed to radiation was compared to that of cells not exposed to radiation (control, 0 Gy). The cell lines or PDOs located in a spot on the pillar were stained with 40 μL of 1 μM calcein AM (Invitrogen) mixed with basal medium for 1 h to visualize 3D tumor cells within the BME. Images of calcein AM-stained cells were acquired using an automated optical fluorescence scanner (ASFA Scanner ST, Medical & Bio Device). Green fluorescent spots observed after scanning indicated the presence of viable cells within the ECM. The intensity of green fluorescence was quantified using an 8-bit scale for each primary color channel.

The viability of the PDOs was also evaluated by calculating the size of each green spot on the CRS™ pillar in Figure 3. The size of each green spot was determined by summing all pixels within the area of a single colony that exhibited an intensity greater than 20 on the code scale. Before irradiation, colonies within a spot were stained, and the initial mean colony area was calculated by dividing the total area by the number of colonies. After the first and second irradiations, the mean colony areas at each radiation dose (0, 2, 4, and 8 Gy) were measured from the final scanned images, as shown in Figures 1B and 3A. In Figures 1C and 3B, growth inhibition is plotted against radiation doses (0, 2, 4, and 8 Gy) using GraphPad Prism 10 (GraphPad Software, Inc.) to generate radiation response curves. Growth inhibition was calculated by comparing colony size at Day 14 relative to control (0 Gy). The GI-AUC values were automatically calculated using the XY analysis function in GraphPad Prism 10.

#### Experimental protocol of histology and immunostaining

Additional quality control (QC) samples were prepared to verify whether the PDOs retained oncological characteristics similar to those of the primary tumor. The prepared PDOs for QC were subjected to IHC analysis similar to the patient tissue (Figure 2B). Isolated PDCs were seeded in 4-well plates at a concentration of approximately 1 × 10^5^ cells and 70% BME (70 v/v) per 30 μL volume. The 4-well plate containing the PDCs was transferred and gelled for 30 min at 37°C in a humidified incubator containing 5% CO_2_. The cells were then cultured for approximately two weeks until PDOs were formed, and the culture medium was changed every four days. The PDOs were fixed, embedded in paraffin, sectioned, and stained. Cytokeratin (CK)5/6, P53, and Ki67 were stained using a Bond-III stainer (Leica Microsystems). Immunohistochemical images were acquired using an Ocus40 digital microscope scanner (Grundium Oy, Finland). The samples were confirmed to be tumor tissues based on histopathological assessment. While the initial evaluation focused on morphological and qualitative similarities, we have now performed a quantitative histological analysis using QuPath (v0.6.0), an open-source digital pathology software. To ensure objectivity, we quantified the expression of CK5/6 using H-scores and evaluated P53 and Ki67 based on the percentage of positive cells. Each slide image was processed using an automated cell detection and classification algorithm according to QuPath’s protocol. The diagnosis of each case was confirmed by pathologists at Samsung Medical Center (Seoul, Korea).

#### Calculation of CODRP indices (multiple logistic regression; MLR analysis)

The CODRP index was calculated by comprehensively considering the cTNM score of Esophageal Cancer (cancer stage), Growth rate (GR), and GI-AUC value converted to a *Z* score (Table 2). For the entire group of 18 patients, we calculated the multiparameter-based CODRP index using the following equation:

(Equation 1) $$CODRP=Z-score\left(\beta_{0}+\beta_{1}X_{1}+\beta_{2}X_{2}+\beta_{3}X_{3}\cdot \cdot \cdot \cdot \cdot \right)$$

where *X*_*1*_*, X*_*2*_*,* and *X*_*3*_ represent independent variables (cancer stage, cell growth rate, and GI-AUC), and constant values (*β*_*0*_*, β*_*1*_*, β*_*2*_*,* and *β*_*3*_) were calculated using the MLR analysis. To predict the pathological response to NCRT, multiple logistic regression parameters were calculated by assigning a value of 0 to a pathological response score of 0, and 1 to all other scores. The CODRP index for pathological responses was determined as follows:

(Equation 2) $${CODRP}^{Pathological\;response}=Z-score\left(1.322+1.849\times Z-score\left(cTNM\right)+3.489\times Z-score\left(GR\right)+2.046\times Z-score\left(GI-AUC\right)\right)$$

To predict recurrence risk, multiple logistic regression parameters were calculated by assigning a value of 0 to cases without recurrence and of 1 to cases with recurrence within 2 years.

The CODRP index for recurrence is calculated as follows:

(Equation 3) $${CODRP}^{Recurrencee}=Z-score\left(1.41+0.8278\times Z-score\left(cTNM\right)+1.634\times Z-score\left(GR\right)+0.07219\times Z-score\left(GI-AUC\right)+0.5108\times Z-score\left(Pathological\;grade\right)\right)$$

In both cases, the cell growth rate demonstrated a significant predictive value compared to that with cTNM and GI-AUC. This indicates that a higher growth rate is associated with a more aggressive tumor behavior, greater resistance to radiotherapy, and an increased risk of recurrence.

#### Definition of clinical outcomes

The pathological response to neoadjuvant chemoradiotherapy was assessed using the Modified Ryan Tumor Regression Grade system, which classifies tumor regression into four grades (0–3) based on the extent of residual viable tumor cells.^19^^,^^20^ Grade 0, indicating a complete pathological response with no viable cancer cells, was categorized as the good pathologic responder group, whereas grades 1–3, representing increasing levels of residual tumor, were collectively classified as the poor pathologic responder group.

Recurrence-free survival (RFS) was defined as the time from radiotherapy initiation to disease recurrence, death from any cause, or the date of the last follow-up.

### Quantification and statistical analysis

Using GraphPad Prism 10 (GraphPad Software, San Diego, CA, USA), unpaired Student’s t-tests or Mann-Whitney U tests were performed to determine the statistical significance of the comparisons between two groups. For comparisons among multiple radiation-dose groups within a cell line, one-way ANOVA was performed. RFS was evaluated using Kaplan–Meier analysis based on the pathological response and the CODRP index, with group differences assessed using the log-rank test. Statistical significance was set at *p* < 0.05. The results are presented as survival curves with 95% confidence intervals. Data are presented as mean ± SD unless otherwise indicated. Cell-based radiation sensitivity data were obtained from technical replicates within a single experiment, whereas each data point in patient-based analyses represents an individual patient. Where applicable, the corresponding figure legends provide the specific statistical test and sample information. Asterisks indicate statistical significance as follows: ∗*p* < 0.05, ∗∗*p* < 0.01, ∗∗∗*p* < 0.001, and ∗∗∗∗*p* < 0.0001. To quantify the robustness of the assay, the Separation Window (SW) was calculated for each dose. Also, the global separation was evaluated using the Discrimination Index (DI). The Z′-factor was additionally calculated for each dose to assess assay quality. In these equations, μ and σ refer to the mean and standard deviation of cell viability, respectively. The subscripts ‘Res’ and ‘Sen’ denote the resistant (SK-GT-4) and sensitive (FLO-1) cell lines. AUC represents the area under the survival curve, and SE indicates the standard error associated with the AUC calculation.

$$SW=\frac{\left|\mu_{Res}-\mu_{Sen}\right|-\left(3\sigma_{Res}+3\sigma_{Sen}\right)}{\sigma_{Res}}$$

$$DI=\frac{\left|{AUC}_{Res}-{AUC}_{Sen}\right|}{\sqrt{{SE}_{Res}^{2}+{SE}_{Sen}^{2}}}$$

$$Z^{\prime }=1-\frac{3\left(\sigma_{Sen}+\sigma_{Res}\right)}{\left|\mu_{Res}-\mu_{Sen}\right|}$$

### Additional resources

This study was not a clinical trial and was not pre-registered. Therefore, no clinical trial registry number or associated link is applicable.
