# Supplementary material for: Patient-derived organoids support radiotherapy response and recurrence risk prediction in esophageal squamous cancer
Source: iScience. 2026 Aug 10;29(8):117100. doi: 10.1016/j.isci.2026.117100 (PMC13486321; doi:10.1016/j.isci.2026.117100)
Supplement: Supplementary file 1 [file mmc1.pdf]

iScience, Volume *29*

Supplemental information

Patient-derived organoids support radiotherapy response and recurrence risk prediction in esophageal squamous cancer

Eunji Jeong, Jong Yun Baek, Sang-Yun Lee, Yongmun Choi, Bosung Ku, Dan Bi Park, Man Ki Chung, Dong Woo Lee, and Dongryul Oh

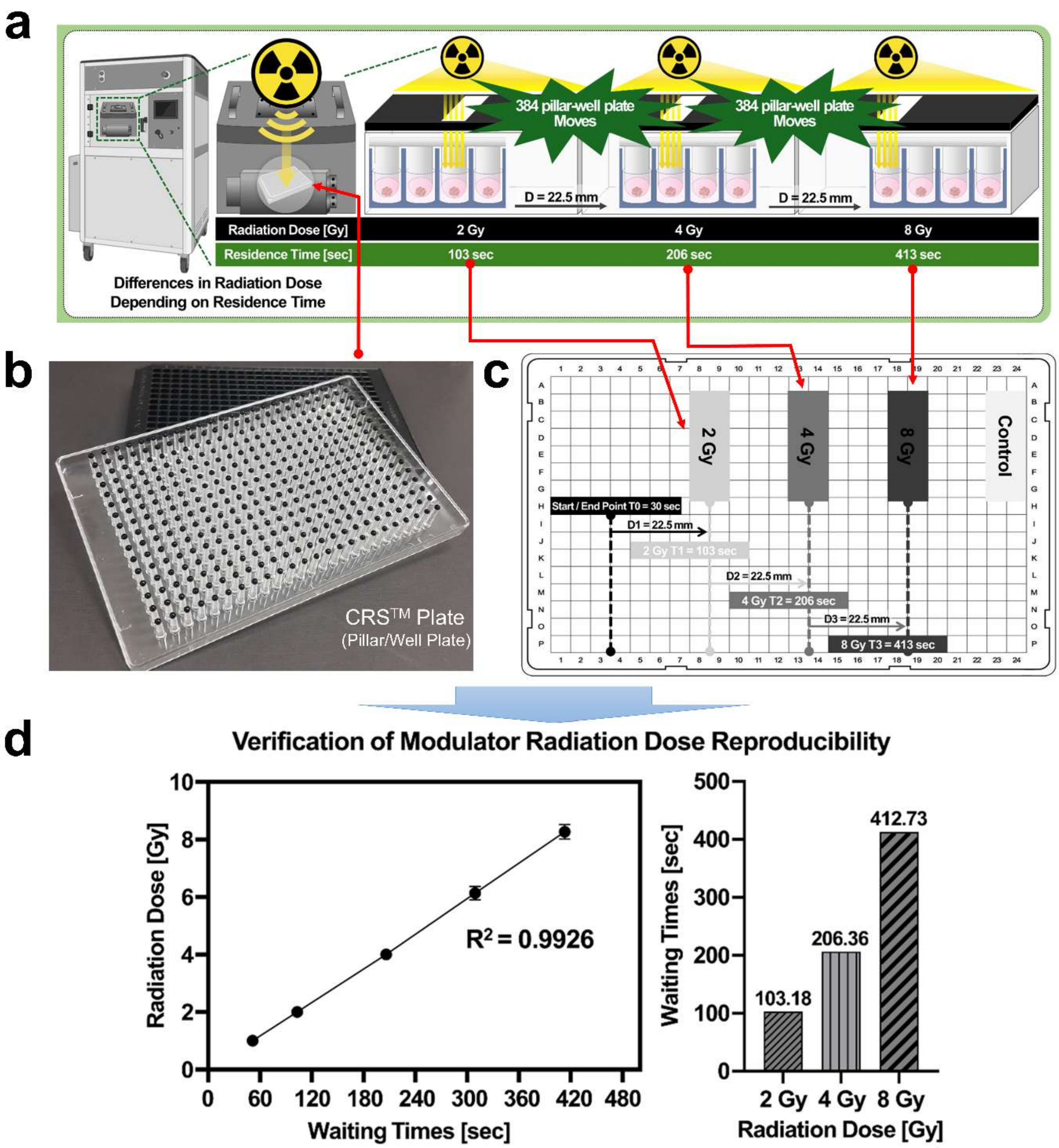

Supplementary Figure. S1: Overview of radiation treatment using the CRS™ Plate. (a) Application of 2 Gy, 4 Gy, and 8 Gy radiation doses by positioning the CRS™ plates within the open area of the slit for specific durations. (b) Fabricated structure of the CRS™ Plate. (c) Schematic layout of radiation exposure across the CRS™ Plate. (d) Quantification of radiation dose relative to exposure duration with linear regression ($R^2 = 0.9926$).

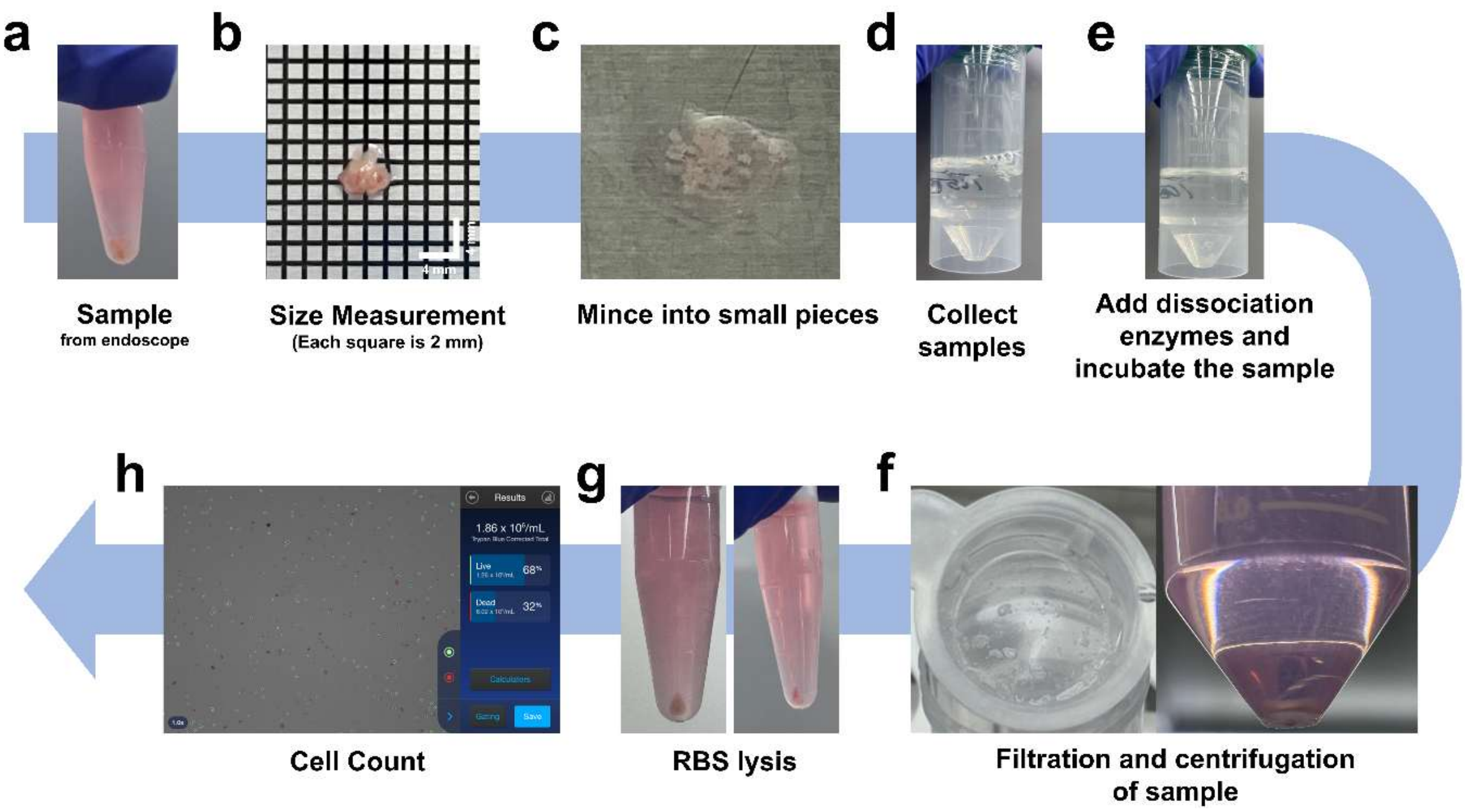

Supplementary Figure. S2: Experimental procedure for isolating patient-derived cells from esophageal cancer tissue. (a) Tissue sample obtained from a patient with esophageal cancer via esophagogastroduodenoscopy. (b) Measurement of tissue sample size. (c) Tissue minced into small pieces. (d) Collection of minced tissue into a tube. (e) Addition of dissociation enzymes to the minced tissue, followed by incubation. (f) Filtration and centrifugation to process the sample. (g) Red blood cell lysis to remove erythrocytes. (h) Counting of live and dead cells using an automated cell counter, with the total viable cell concentration recorded.

Supplementary Table S1. Raw QuPath-derived IHC quantification of matched primary tumors and patient-derived organoids

No	ID	Sample type	CK5/6 H-score	p53-positive cells (%)	Ki-67-positive cells (%)
1	RTS278	Primary tumor	291.1	0.3	27.7
		PDO	237.3	10.7	35.3
2	RTS288	Primary tumor	135.2	41.7	33.9
		PDO	187.0	32.2	21.9
3	RTS312	Primary tumor	254.0	2.2	10.6
		PDO	197.9	6.7	20.5
